# Comparative research on ^99m^Tc-Rituximab and ^99m^Tc-sulfur colloid in sentinel lymph node imaging of breast cancer

**DOI:** 10.1186/s12885-019-6197-9

**Published:** 2019-10-15

**Authors:** Jing-Jie Zhang, Wan-Chun Zhang, Cai-Xia An, Xiao-Min Li, Le Ma

**Affiliations:** 1 Department of nuclear medicine, Hebei General Hospital, Shijiazhuang, China; 2 Department of nuclear medicine, Shanxi Academy Of Medicial Sciences, Shanxi Dayi Hospital, Taiyuan, China

**Keywords:** Breast cancer, Sentinel lymph node mapping, Radiopharmaceuticals, CD20

## Abstract

**Background:**

^99m^Tc-Rituximab is a new specific radiopharmaceutical that binds to the CD20 receptor which is highly expressed on the surface of B cells. We conducted a study in which ^99m^Tc-Rituximab was compared with filtered ^99m^Tc-sulfur colloid (fTcSC) for sentinel lymph node (SLN) detection in patients with breast cancer.

**Method:**

The study is divided into three parts. 1. Initially, 25 patients were selected for an internal controlled trial to received both ^99m^Tc-Rituximab and fTcSC, the interval time is separated by ≥2 days. 2. Then, 91 patients were selected for a randomized controlled trial (41 and 50 patients in the ^99m^Tc-Rituximab and fTcSC groups, respectively). All patients were administered either agent at the 6- and 12-o’ clock positions by subareolar injection technique. SLN mapping was then performed 2 h after injection. 3. Serial dynamic images were further acquired for 2 h in 31 patients (22 and 9 patients from ^99m^Tc-Rituximab and fTcSC cohorts, respectively).

**Results:**

The identification rate of lymphoscintigraphy and SLNB in all and axilla regions for ^99m^Tc-Rituximab and ^99m^Tc-SC were 98.5% vs 98.7, 100% vs 98.4%, respectively. The mean number of SLNs identified by ^99m^Tc-Rituximab and fTcSC was respectively 2.72 and 3.28, with a significant difference of *P* = 0.013 (paired sample t-test). The difference exists in the internal mammary and clavicular area, not in the axillary. The mean number of axillary sentinel lymph node biopsy (SLNB) for ^99m^Tc-Rituximab and fTcSC was 2.95 vs 3.14, respectively, and no significant difference existed. ^99m^Tc-Rituximab also exhibited a significantly faster injection site clearance rate when compared with fTcSC (0.193 ± 0.057 h^− 1^ vs 0.021 ± 0.007 h^− 1^, respectively).

**Conclusion:**

No significant difference was observed in identification rate and number of axillary SLN imaging and SLNB, between the two tracers. Compared to fTcSC, ^99m^Tc-Rituximab based imaging demonstrated a fewer number of secondary lymph nodes and had faster injection site clearance rate.

**Trial registration:**

www.chictr.org.cn, ChiCTR1900024990 (retrospectively registered August 6, 2019).

## Background

Axillary nodal involvement in breast cancer has a significant impact on tumor staging, treatment, and prognosis. The concept of SLN, first described in 1977 by Cabanas and colleagues, and is defined as the first lymph node that receives afferent lymphatic drainage from a primary tumor [1]. For early breast cancer diagnosis, SLNB can be used in lieu of axillary lymph node dissection (ALND) to assess the metastasis status of the axillary lymph nodes (ALN) and also to reduce morbidity [2].

Despite the widespread use of SLNB for breast cancer, there is still uncertainty in regards the optimal agent for SLN identification. Radiopharmaceuticals and vital blue dye (VBD) are now commonly used. The most common agent used in the United States is ^99m^Tc-sulfur colloid (^99m^Tc-SC), whereas, in Europe, the most used tracer is ^99m^Tc-HSA nanocolloid [3]. Still, none of these agents have been designed for SLN detection and, therefore, a more in-depth evaluation is further required. In principle, the major criterion for the evaluation of an imaging agent is the particle size. Optimal particle size can ensure rapid clearance rate from the injection site, high retention in SLN, and low accumulation in distal nodes.

An ideal radiopharmaceutical for SLN detection should exhibit rapid clearance from the injection site, rapid uptake, high retention within the first draining lymph node, and low uptake by distal lymph nodes. These characteristics can be fulfilled based on the properties of novel ligand-receptor or antigen-antibody specific binding agents for SLN mapping [4]. Lymphoseek™, a radiopharmaceutical specifically designed for SLN detection, is known to accumulate in lymphatic tissues by binding to CD206, a mannose receptor that resides on the surface of macrophage cells [5], and has been approved by the US Food and Drug Administration (FDA) in 2013. Studies focusing on Lymphoseek have shown that this molecule meets the requirements of an ideal tracer.

CD206 and CD20 are current research focuses of tracer targets for tracer development in regards SLN agents. The CD20 antigen is specifically highly expressed at the surface of almost all normal and malignant B cells, and is neither shed from the cell surface nor internalized after binding to specific antibodies [6]. It is also the target of Rituximab, one of the most effective antitumor monoclonal antibodies developed so far. Xuejuan Wang et al. [7] has reported the preparation and use of ^99m^Tc-Rituximab for SLN detection in animal models and confirmed that it exhibits the desired properties of rapid clearance from the injection site as well as low accumulation in distal nodes. In this work, we carried out a clinical trial to confirm these characteristics in human subjects and to further compare the detection efficiency between ^99m^Tc-Rituximab and fTcSC.

## Methods

### Patients and groups

Patients diagnosed with breast cancer who underwent SLNB, in the period of Oct 2016 to Oct 2017, participated in this study. Procedures were followed according to the EANM and SNMMI practice guidelines [8]. The inclusion criteria included clinical lymph node-negative breast cancer and patients who received neoadjuvant chemotherapy. Cases with suspicious, palpable axillary nodes and inflammatory breast cancer were excluded. The Shanxi DaYi Hospital institutional review board (IRB) approved this study, and all patients signed an informed consent form.

Clinical study was divided into three parts, all composed by simple randomized grouping trials. Firstly, 25 patients in an internal-controlled trial, were randomly divided into 2 groups. Thirteen patients received ^99m^Tc-Rituximab at first, and fTcSC 2 days later. Twelve patients first received fTcSC, and followed by ^99m^Tc-Rituximab 2 days later. Secondly, 91 patients participated in a randomized controlled trial, and there were 41 patients in the ^99m^Tc-Rituximab group and 50 patients in the fTcSC group. Thirdly, 31 subjects participated in the injection site clearance rate trial. And 22 versus 9 patients were administered with ^99m^Tc-Rituximab versus fTcSC, respectively. Serial dynamic images were acquired for 2 h. The age range of the subjects was between 32 to 76 years old (mean: 51.09 ± 9.11 years old; Table 1). Lesion size ranged from 0.5 to 6 cm (mean: 2.35 ± 1.10 cm). Additional clinical data are disclosed in Table 1.

**Table 1:** Clinical data of all patients included in the trial

	Internal-controlled trial	Randomized controlled trial		
	No.of patients	No. of patients		
		^99m^Tc-Rituximab	fTcSC	Significance
Age (years)				
≤ 50	16	22	23	*P* = 0.467
> 50	9	19	27	
Breast type				
dense breast	12	22	24	*P* = 0.591
non-dense breast	13	19	26	
Tumor size (cm)				
≤ 2	14	15	26	*P* = 0.071
2 < R ≤ 5	11	23	24	
> 5	0	3	0	
Pathological type				
ductal carcinoma in situ	3	9	6	*P* = 0.236
infiltrating ductal carcinoma	21	28	41	
special type	1	2	3	
mixed type	0	2	0	
Tumor location				
Left breast	12	23	23	*P* = 0.338
Right breast	13	18	27	
Upper outer quadrant	17	20	28	*P* = 0.265
Lower outer quadrant	2	7	8	
Upper inner quadrant	4	8	9	
Lower inner quadrant	2	6	2	
Center	0	0	3	
Molecular type				
Luminal A	2	3	6	*P* = 0.802
Luminal B	11	20	27	
ERBB2+	6	5	6	
Basal-like	5	8	8	
Deletion	1	5	3	

### Agent preparation and administration method

^99m^Tc-Rituximab was synthesized and radiolabeled according to the 2-mercaptoethanol (2-ME) method, described by Li Nan [9], at the Peking University Cancer Hospital. Radiochemical purity was > 92%. Injection dose of ^99m^Tc-Rituximab was standardized as 74 MBq in 1 ml on the day before operation, and 37 MBq in 1 ml on the operative day. The fTcSC was radiolabeled, boiled for 3 min, and passed by a 200 nm membrane for filtration, and the radiochemical purity was always > 91%. The injection dose of fTcSC was 74 MBq in 1.5 ml on the day before operation, and 37 MBq in 1.5 ml on the operative day.

### Nuclear imaging

Each patient was administered with an imaging agent at the 6- and 12-o’clock positions, by applying the subareolar technique with both subcutaneous and intra-parenchymal injection, regardless of the site of breast cancer. Administration site was then massaged for several minutes. Two hours later, a lymphoscintigram was acquired, including planar imaging and fusion tomography, in order to localize the SLNs, which is divided into three areas: ALN, internal mammary lymph nodes (IMLN) and clavicular lymph nodes. The SPECT (GE Discovery NMCT 670) acquisition conditions were set as matrix, 128 × 128; zoom, 1; peak energy, 140 keV; window width, 20%.

For the third group of patients (*n* = 31), dynamic imaging of the injection site was immediately performed after the injection, at 30-min intervals for 2 h. All images (256 × 256) were acquired for 3 mins. The standard source was placed on the level of the contralateral clavicle of the breast lesions. The clearance rate constant (kc) and half-life (Tc) at the injection site were calculated using decay corrected counts, obtained from the nuclear images of the injection site.

### Surgical technique

Both blue dye node localization and radioguided surgery were employed for SLNB. Methylene blue (MB), injected by subareolar and subcutaneous technique, was used to trace blue-stained nodes. During operation, γ detector identified all nodes with counts rate greater than 10% of the nodes with the highest count rate, that is, hot nodes [10]. Blue dye nodes and hot nodes were defined as SLNs. The intumescent, hard and suspicious lymph nodes were also excised as SLNs.

### SLN pathology

All excised lymph nodes were examined by intraoperative frozen section and printing slice cytology analysis. Classification of SLN metastases followed according to the 8th edition of AJCC, and in our study a positive SLN was identified, when it contained metastatic tumors with sizes > 0.2 mm, and then the lymph node basin was completely dissected.

### Statistical analysis

Chi square analysis or Fisher’s exact test was used to compare clinical data, tumor features, and SLN identification rates between groups. The Wilcoxon rank-sum statistical test was also used to compare the number of SLNs detected by the two agents. T-test was applied to calculate significant differences in the kc and Tc values between the ^99m^Tc-Rituximab and the fTcSC. A difference with a *P* value < 0.05 was considered statistically significant.

## Results

### Internal-controlled trial

Twenty-five patients were enrolled in the internal-controlled trail in which paired injections of each agent were administered, at separate times and on different days. And lymphoscintigraphies were compared. Both imaging agents achieved a high SLN imaging identification rate of 96%. Both tracers failed to detect SLNs in one patient who performed SLNB after neoadjuvant chemotherapy. No statistical difference was observed in the identification rate of ALN and IMLN, detected between ^99m^Tc-Rituximab and fTcSC. As for clavicular lymph nodes, the identification rate detected by ^99m^Tc-Rituximab was 8%, which was less than fTcSC, 28%. Still, this difference was not statistically significant, due to the small sample size.

**Fig. 1:**
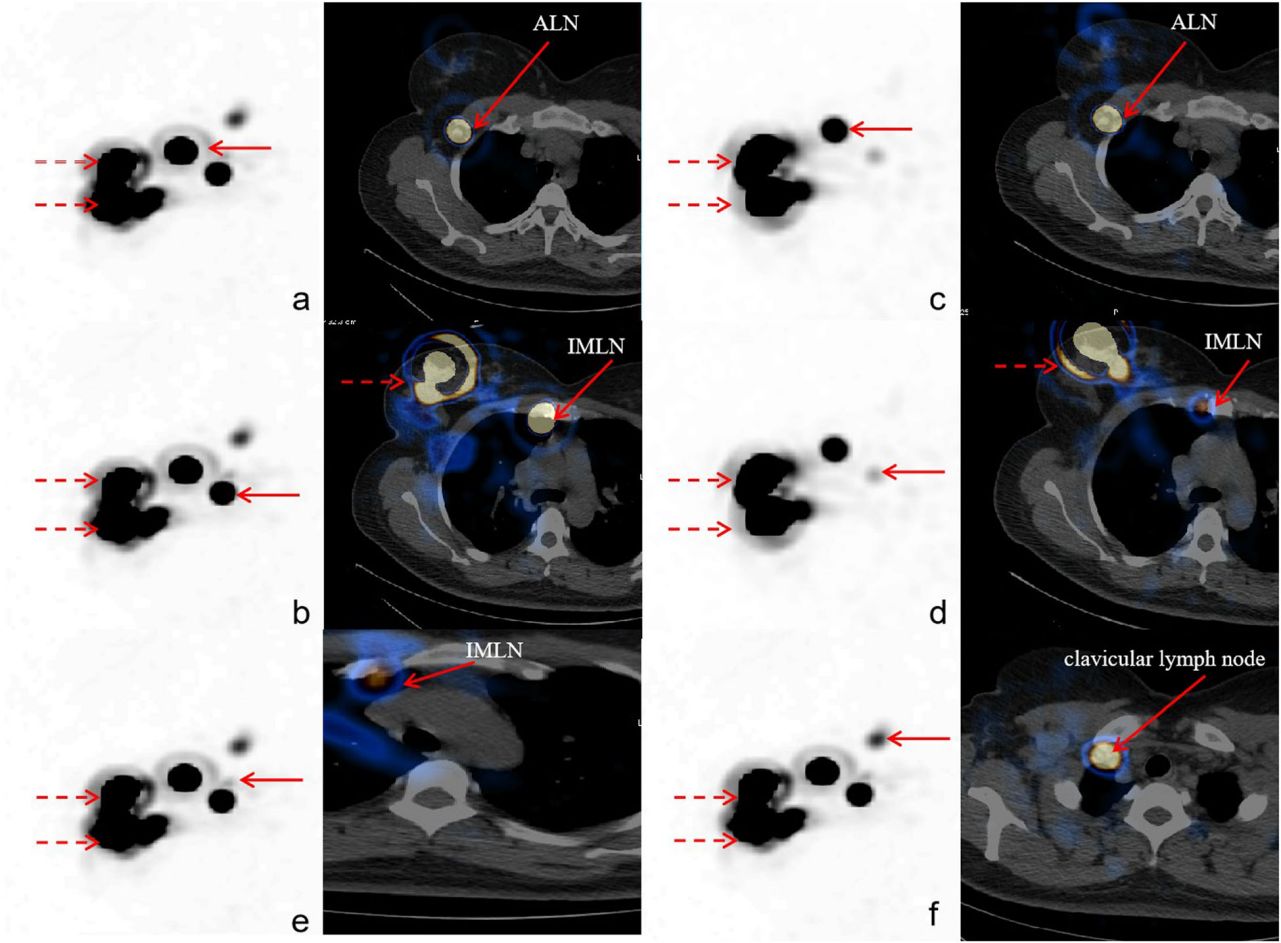
Dotted line represents the injection site, and the solid line represents SLN. **a** and **b**: patient injected with ^99m^Tc-Rituximab (37 MBq in 1 ml); 2 SLNs were detected; **c**-**f**: patient injected with fTcSC (37 MBq in 1.5 ml), 4 SLNs were detected. The SLNs shown in figure panels (**a**) and **c**, **b** and **d** were detected the same location. The extra SLNs marked in panels (**e**) and **f** correspond to IMLN and clavicular lymph node, respectively

The total number of SLNs identified by ^99m^Tc-Rituximab and fTcSC were respectively 68 vs 82, (mean 2.72 vs 3.28), and the difference had statistical significance (*P* = 0.013). ^99m^Tc-Rituximab identified fewer or the same number of SLNs, while all of them were detected by fTcSC, with a 82.9% concordance rate (68/82). There was no statistical difference in the average number of ALN detected between ^99m^Tc-Rituximab and fTcSC (total 58 vs 61, mean 2.32 vs 2.44, respectively; *P* > 0.05). A significant difference between the two agents was observed in the number of both IMLN and clavicular lymph nodes, and the total number of SLNs identified by ^99m^Tc-Rituximab or fTcSC was 8 vs 12, 2 vs 9, respectively. Figure 1 shows a representative image of a fusion tomography of SLNs acquired from one patient, where ^99m^Tc-Rituximab detected 2 SLNs while fTcSC detected 4 SLNs.

### Randomized controlled trial

There was no significant difference in age, breast type, tumor size, pathological type, tumor location and molecular typing between the two groups (P > 0.05). The identification rate of SLNs imaging and SLNB was 100% for both groups. The mean number of SLNs detected by imaging with ^99m^Tc-Rituximab and fTcSC tracers was 2.61 and 2.92, respectively, whereas the number detected by SLNB was 2.95 and 3.13, respectively. The metastatic rate for ^99m^Tc-Rituximab and fTcSC was 17.07 and 20%, and no statistically significant difference existed between the two agents. Also, no significant difference between ^99m^Tc-Rituximab and fTcSC was observed in the directive function of operation. We found that fusion tomography for SLN localization, combined with ^99m^Tc-Rituximab, had lower identification rate and detected fewer numbers of clavicular lymph nodes than fTcSC, and this difference was statistically significant (*P* < 0.05).

**Fig. 2:**
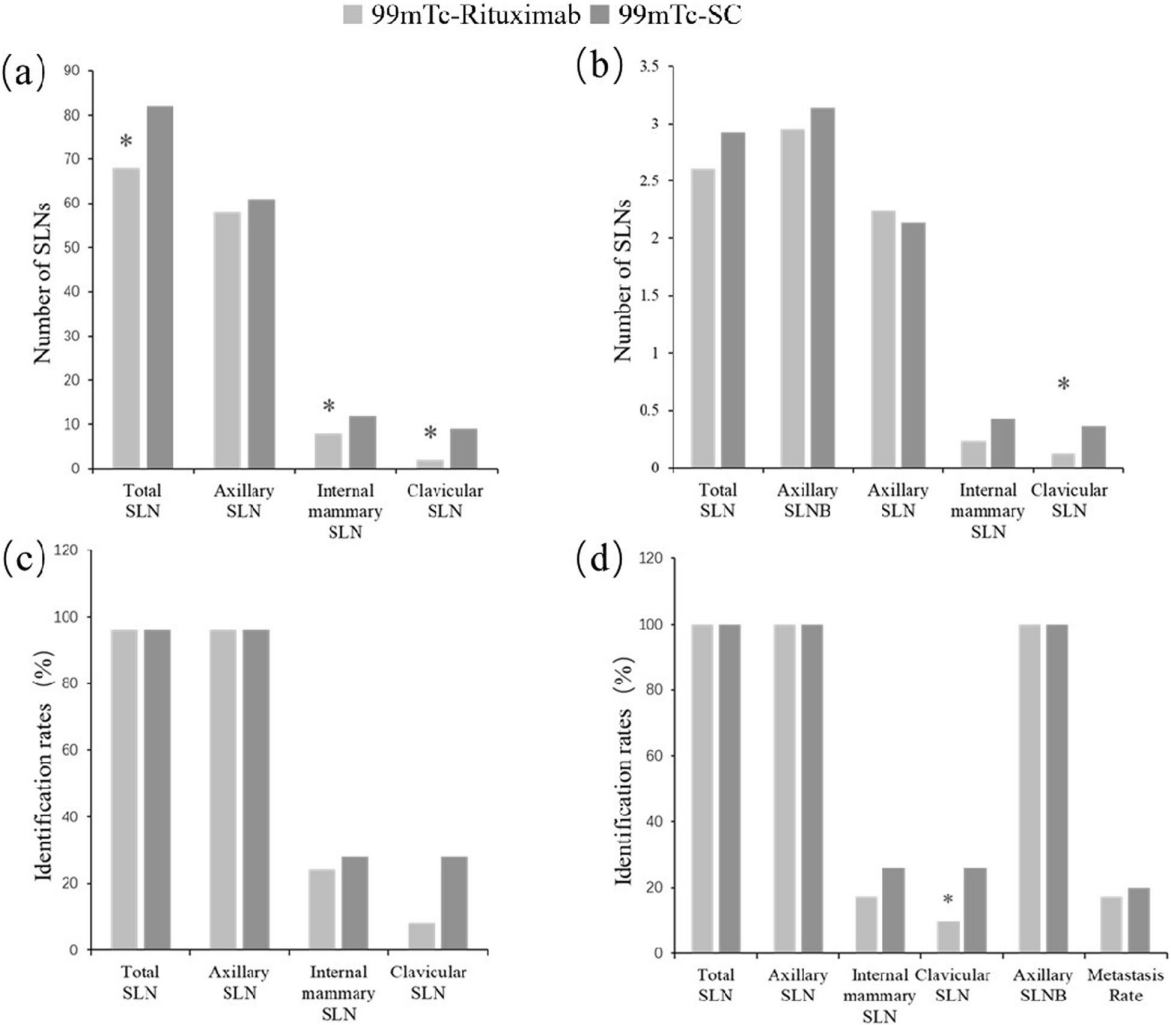
SLN counting and identification rates in different regions. **a** and **c** show the results of the internal-controlled trial, while **b** and **d** belong to a randomized controlled trial. * *p* < 0.05 was used as a cut-off for the significant difference between the two agents

SLN counting and identification rates of the two former trails are shown in Fig. 2. We found that the two agents had similar identification rate of SLN, ALN and IMLN. In regards clavicular lymph nodes, the identification rate of with ^99m^Tc-Rituximab was less than fTcSC, but only in randomized controlled trial, the difference was significant. In addition, we have found that the both agents had similar numbers of ALN and SLNB. ^99m^Tc-Rituximab had fewer number of total SLN, IMLN in internal-controlled trial and fewer clavicular lymph nodes in the two former trials. Although the same items of those two former trails have similar value, their difference was not significant possibly due to the small cohort patients.

### Injection site clearance rate trial

**Fig. 3:**
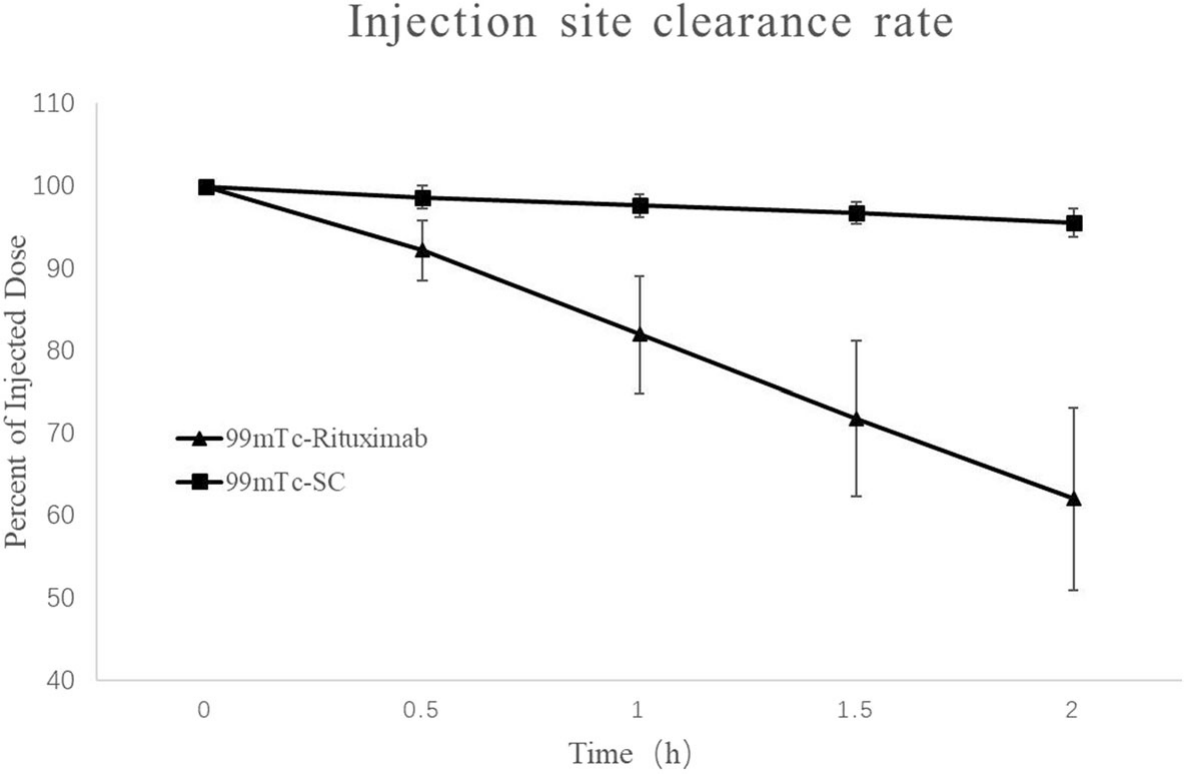
The mean injection site clearance rates of ^99m^Tc-Rituximab and fTcSC. The clearance rate constant (kc) for each tracer was calculated from the slope of the corresponding graph line. The (kc) for ^99m^Tc-Rituximab (solid triangle) was 0.193 ± 0.057 h^− 1^, and for fTcSC (solid square) was 0.021 ± 0.007 h^− 1^

We have observed that ^99m^Tc-Rituximab exhibited a significantly faster injection site clearance rate and shorter half-life. The clearance rate constants for ^99m^Tc-Rituximab ranged from 0.070 to 0.309 h^− 1^ and from 0.012 to 0.035 h^− 1^ for fTcSC. The mean injection site clearance rate constant (kc) for ^99m^Tc-Rituximab was 0.193 ± 0.057 h^− 1^, which was higher (about 10 times) than the mean (kc) for fTcSC, which was 0.021 ± 0.007 h^− 1^. The acquired (Tc) value for ^99m^Tc-Rituximab was 3.996 ± 1.600 h and 36.053 ± 12.495 h for fTcSC. Figure 3 illustrates the mean injection site clearance rate constant of ^99m^Tc-Rituximab and fTcSC.

## Discussion

SLN is the first lymph node to receive drainage from a lymphatic tumor. Ideally, only the anatomical SLNs should be excised. In fact, unnecessary removal of secondary lymph nodes can result in excessive dissection, which increases the incidence of seromas and infection [11]. Since breast lymphatic drainage is complex, it is usually difficult to distinguish between SLN and secondary lymph nodes. In internal-controlled trial, we have found that fTcSC identified more SLNs when compared to ^99m^Tc-Rituximab. All patients underwent fusion tomography to accurately locate SLNs, and it has been found that the secondary lymph nodes detected by fTcSC were mainly located in the internal mammary and clavicular basin, rather than in the axillary region. Breast lymph is mainly drained to the axillary nodes, then successively passed to subclavian and supraclavicular lymph nodes, which means that clavicular lymph nodes correspond to distal lymph nodes. The study of Cao XS et al. [12] on the lymphatic drainage system in breast cancer showed that lymph from different breast areas drain into the same IMLN, which is then transferred to other IMLNs, therefore indicating that the remaining nodes can be classified as secondary lymph nodes. FTcSC identified more IMLN and clavicular lymph nodes, which indicated that it had lower specificity and, therefore, detected more secondary lymph nodes as SLNs.

The principal mechanism of action of most targeting agents is mediated by the specific binding to ligands and their receptors, so when a SLN is not saturated, the agents are unable to drain to the secondary lymph nodes. This is due to the fact that, only under SLN saturating conditions, distal nodes will be able to uptake the agents. Consequently, under optimal dose administration, these agents can target and further identify fewer secondary lymph nodes. This phenomenon has also been demonstrated in some studies related to Lymphoseek [13, 14].

We compared breast cancer patients undergoing axillary SLNB who received ^99m^Tc-Rituximab+VBD or fTcSC+VBD. No significant difference between these groups was observed, in regards the directive function of operation. A preliminary clinical study, carried by Li Nan and colleagues, revealed that ^99m^Tc-Rituximab have a high success rate (100%) and a low false negative rate (2.60%) [9]. The guideline of the American Society of Clinical Oncology recommends an identification rate of 85% with a false-negative rate of 5% or less for SLNB [15]. In this study, the success rate of SLNB in all patients, who received radioactive tracers before operation and VBD during operation, was 100%. Moreover, the average number of SLNs here detected by imaging with ^99m^Tc-Rituximab and SLNB was 2.61 and 2.95, respectively. Compared to a larger sample clinical trial [9], the corresponding numbers were 1.78 and 2.85. The yield numbers in preoperative SLN imaging were similar to the values obtained by the intraoperative SLNB. This may be due to the fact that hot spot SLNs are possibly restricted to regions other than the injection site, and that fusion tomography may detect more lymph nodes than planar imaging.

Although the excision of a lower content of SLNs may reduce the surgical time and treatment costs, the detection accuracy of SLNB is essential. The minimal number of SLNs that are necessary for accurate axillary staging has not been determined yet [16]. Many researchers have shown that removing more lymph nodes can achieve an acceptable false negative detection rate for SLNB [17]. In this regard, relevant studies on ^99m^Tc-Rituximab have not been previously reported.

Here, we demonstrate that ^99m^Tc-Rituximab exhibits a significantly faster injection site clearance rate and shorter half-life than fTcSC. This indicates low residue and scattering at the injection site, which can reduce the interference with precise SLN identification, especially when SLN is located near the injection point. In addition, the low scattering effect of ^99m^Tc-Rituximab could potentially prevent unnecessary radiation exposure to the surgical team.

## Conclusion

Compared with fTcSC, the antigen-antibody binding agent ^99m^Tc-Rituximab has equivalent detection rates for ALN and IMLN, as well as similar operation guidance of axillary SLNB, leading to fewer non-SLNs and faster clearance at the injection point. These features define ^99m^Tc-Rituximab as a potential specific tracer, therefore providing a novel and clinically valuable approach for SLNB.

## Data Availability

The datasets used and analyzed in the current study are available from the corresponding author on reasonable request.

## Abbreviations

^99m^Tc-SC: ^99m^Tc-sulfur colloid
ALN: axillary lymph nodes
ALND: Axillary lymph node dissection
fTcSC: filtered ^99m^Tc-sulfur colloid
IMLN: Internal mammary lymph nodes
SLN: Sentinel lymph node
SLNB: Sentinel lymph node biopsy
VBD: Vital blue dye
